# Intra-Accumbens Injection of a Dopamine Aptamer Abates MK-801-Induced Cognitive Dysfunction in a Model of Schizophrenia

**DOI:** 10.1371/journal.pone.0022239

**Published:** 2011-07-13

**Authors:** Matthew R. Holahan, Dan Madularu, Erin M. McConnell, Ryan Walsh, Maria C. DeRosa

**Affiliations:** 1 Department of Neuroscience, Carleton University, Ottawa, Ontario, Canada; 2 Department of Chemistry, Carleton University, Ottawa, Ontario, Canada; University of New South Wales, Australia

## Abstract

Systemic administration of the noncompetitive NMDA-receptor antagonist, MK-801, has been proposed to model cognitive deficits similar to those seen in patients with schizophrenia. The present work investigated the ability of a dopamine-binding DNA aptamer to regulate these MK-801-induced cognitive deficits when injected into the nucleus accumbens. Rats were trained to bar press for chocolate pellet rewards then randomly assigned to receive an intra-accumbens injection of a DNA aptamer (200 nM; n = 7), tris buffer (n = 6) or a randomized DNA oligonucleotide (n = 7). Animals were then treated systemically with MK-801 (0.1 mg/kg) and tested for their ability to extinguish their bar pressing response. Two control groups were also included that did not receive MK-801. Data revealed that injection of Tris buffer or the random oligonucleotide sequence into the nucleus accumbens prior to treatment with MK-801 did not reduce the MK-801-induced extinction deficit. Animals continued to press at a high rate over the entire course of the extinction session. Injection of the dopamine aptamer reversed this MK-801-induced elevation in lever pressing to levels as seen in rats not treated with MK-801. Tests for activity showed that the aptamer did not impair locomotor activity. Results demonstrate the *in vivo* utility of DNA aptamers as tools to investigate neurobiological processes in preclinical animal models of mental health disease.

## Introduction

In general, schizophrenia is characterized by positive, negative and cognitive symptoms. Cognitive deficits appear in the realm of working memory, executive function, attention and processing speed [1]. One aspect of executive function concerns updating, or monitoring, information in one's working memory, which has been considered in terms of coding incoming information and replacing what is no longer relevant [2]. The concept of inhibition contributes to this aspect of executive function where there is a requirement to intentionally overrule a propensity to make an automatic, predominant response. At the heart of this is the ability of an organism to switch behavioral output in response to a change in environmental contingencies [3]. Individuals with schizophrenia are said to ‘perseverate’, showing difficulty in inhibiting behavioral tendencies on a previously learned task even when it is inappropriate to do so [4].

NMDA receptor antagonists, such as MK-801, have been used in rodents to model cognitive dysfunction as would be observed in individuals with schizophrenia [5]–[6]. In one behavioral model using MK-801, rats are trained to press a lever to receive a chocolate pellet reward. After 5 days of training, rats are subjected to one extinction test, whereby lever pressing no longer results in presentation of a chocolate pellet reward. In normal rodents, during the extinction test, the lever pressing response starts high but quickly decreases (extinguishes) showing that the rats are able to inhibit their behavioral tendency to press the lever. Work has shown that a moderate dose (0.05–0.1 mg/kg) of MK-801 produces a persistent, elevated lever pressing response during the extinction test [7]–[8] modeling an executive function deficit (perseveration). In the Holahan, et al., study, D1- or D2-like receptor antagonists reversed the MK-801-induced behavioral profile and the nucleus accumbens showed elevated neural activity in the MK-801-treated group compared to saline controls (8). These data were interpreted to suggest that MK-801 induced cognitive deficits similar to perseveration, which may be in part due to overactivity of dopamine in the nucleus accumbens.

The purpose of the present study was to inject a DNA aptamer with binding affinity for dopamine into the nucleus accumbens and determine its effect on the MK-801-induced deficit in extinction responding. Aptamers are single stranded DNA or RNA sequences that fold into distinct conformations capable of binding to a target molecule [9]. As molecular recognition probes, aptamers have binding affinities and specificities that are comparable to, and in some cases even surpass, those of monoclonal antibodies. In certain applications, aptamer technology can offer several advantages over antibodies [10]. High-purity aptamers can be chemically synthesized at a low cost with no batch-to-batch variability. Aptamers are more chemically stable than antibodies under most conditions, have a longer shelf life, and show little to no immunogenicity. Furthermore, the process for their selection, known as SELEX, is an *in vitro* screening process, which allows for a greater degree of control over the ultimate selectivity and affinity of the final product [11]–[12]. As a result of these advantages, aptamers have emerged as a viable alternative to antibodies in many analytical, diagnostic and therapeutic applications.

Despite their exciting potential, the research surrounding the application of aptamers in the central nervous system is extremely limited [13]. Aptamers have been selected that target B1-CT, the short cytoplasmic tail of the BACE1 protein and βA4, an amyloid peptide, both of which may provide diagnostic or therapeutic tools for Alzheimer's disease [14]. A group of aptamers that competitively binds to Nogo-66 receptor *in vitro* has shown promise in promoting axonal elongation [15]. A DNA aptamer has also been developed to detect Neuropeptide Y, a central nervous system peptide implicated in feeding behaviors [16]. RNA aptamers have been selected that displace cocaine from the nicotinic acetylcholine receptor in cell culture [17]–[18]. Recently, an aptamer-gold nanorod assay was developed to detect adenosine phosphates in samples extracted from the brains of Sprague-Dawley rats [19].

**Figure 1:**
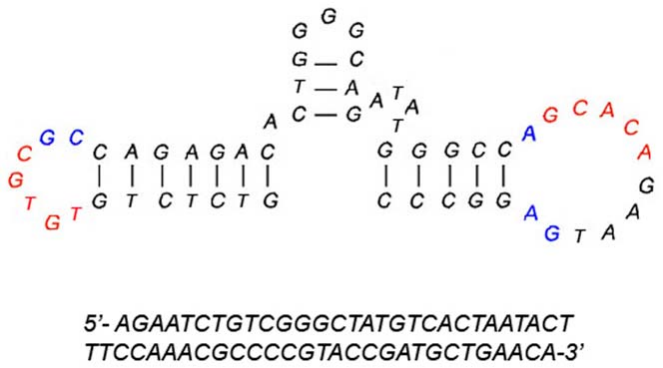
Aptamer sequence. Schematic of the aptamer sequence used in this experiment (top). The two complementary regions shown in red and the conserved bases shown in blue are thought to form the target binding site. Also shown is the sequence of the random oligonucleotide control (bottom).

An RNA aptamer for dopamine with a moderate binding affinity was reported in the late 1990s [20]. More recently, the DNA homolog of this RNA aptamer (see Figure 1 for the sequence) was found to target dopamine with improved binding and stability, and could also bind norepinephrine with a similar affinity [21]. To date, neither the RNA nor DNA dopamine aptamer has been investigated *in vivo*, nor has an aptamer for any target ever been tested directly in the brain.

The primary goal of this work was to investigate whether this DNA dopamine aptamer would retain its binding properties *in vivo*, by injecting it into the nucleus accumbens (Acb) of the rat brain. We hypothesized that the aptamer would work in a similar fashion as an antagonist but, rather than binding to the receptor, bind directly to both dopamine (and norepinephrine) molecules, thus stopping their action on the receptor. In this study, we report on the behavioral effects of aptamer injection into the nucleus accumbens of MK-801-treated rats compared to rats treated with buffer or a random DNA oligonucleotide. Our results suggest that aptamer treatment was selective in mitigating the behavioral effects of peripheral MK-801 administration.

## Results

### Operant Task: intra-accumbens aptamer ameliorates MK-801-induced perseveration

#### Acquisition

Rats were trained drug-free over 5 days to press a lever to receive a chocolate pellet reward. A two-way ANOVA (day by group) on the mean number of correct presses per day revealed a main effect of day (F(4,16) = 94.44, p<0.001) with no group differences (F(4,26) = 1.57) and no interaction (F(16,104) = 1.27). This suggests that all groups learned the task similarly over the 5-day acquisition period which was to be expected as no experimental manipulations were carried out during this time.

#### Extinction

Forty-eight hours after the last rewarded acquisition day, rats were treated with intra-accumbens injection (0.5 µl per hemisphere for all intra-accumbens injections) of tris buffer and systemic MK-801 (0 nM/ MK, n = 7; all MK-801 doses = 0.1 mg/kg/ml), intra-accumbens injection of 200 nM aptamer and systemic MK-801 (200 nM/ MK, n = 7), intra-accumbens injection of the random oligonucleotide sequence and systemic MK-801 (Random/ MK, n = 7), intra-accumbens injection of tris buffer and systemic saline (0 nM/ Saline, n = 5), or intra-accumbens injection of 200 nM aptamer and systemic saline (200 nM/ MK, n = 5). Fifteen minutes after the systemic injection, rats were placed into the operant chambers and given a 30 min extinction session where lever presses did not produce any chocolate pellet reward. Supplemental Videos S1 and S2 show a 30-sec sample of bar pressing behavior from a rat injected with vehicle into the nucleus accumbens and MK-801 systemically (Video S1) and a rat injected with aptamer into the nucleus accumbens and MK-801 systemically (Video S2). The vehicle animal is shown to press incessantly in the absence of reward on the correct lever while the aptamer animal presses a few times then stops when no reward delivery occurs. Both video clips showing pressing behavior at approximately the 20 min mark of the 30 min extinction test.

**Figure 2:**
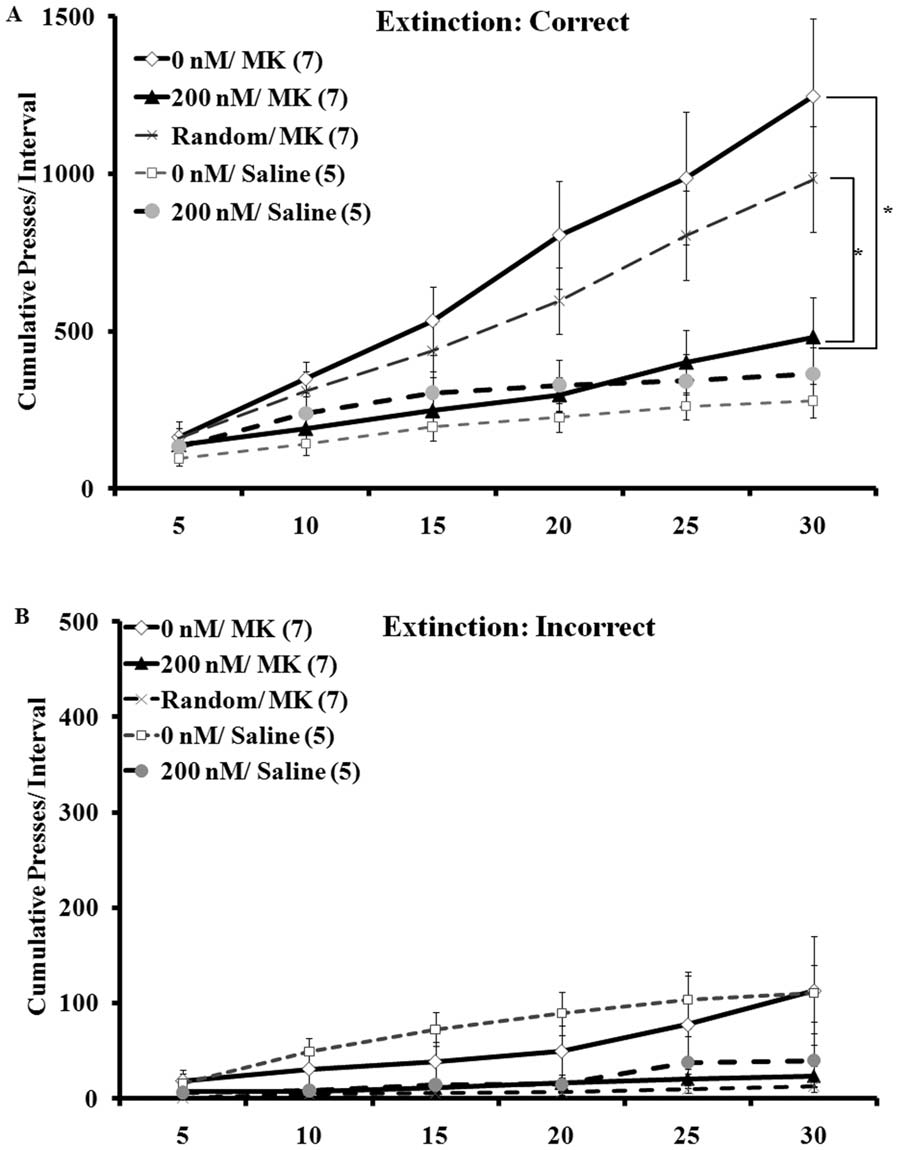
Intra-accumbens aptamer injection reverses the MK-801-induced elevation in extinction pressing. A) Cumulative correct lever responses per 5-min interval over the 30-min extinction session. B) Cumulative number of incorrect lever presses per 5-min interval over the 30-min extinction session. Groups pretreated with intra-accumbens vehicle (0 nM/ MK; n = 7) or the random oligonucleotide sequence (Random/ MK; n = 7) and systemically with 0.1 mg/ kg MK-801 showed elevated correct lever pressing throughout the entire 30-min extinction session compared to animals pretreated with 200 nM dose of the aptamer (200 nM/ MK; n = 7) and both groups treated systemically with saline (0 nM/ saline; n = 5 and 200 nM/ saline; n = 5). Correct extinction pressing was not significantly different between the 200 nM/ MK group and the 0 nM/ saline or the 200 nM/ saline groups. *, p<0.05; numbers in parentheses = animals per group. No significant differences were detected when measuring incorrect lever presses during the extinction session. Data expressed as mean ± SEM.

The extinction data for correct lever presses are shown in Figure 2A. Cumulative correct presses during the extinction test were analyzed with a two-way, repeated-measures ANOVA (group by 5-min time interval). Analysis revealed main effects of group (F(4,26) = 3.84, p<0.05) and interval (F(5,20) = 56.74, p<0.001) and a significant interaction between interval and group (F(20,130) = 6.47, p<0.001). Fisher's Least Significant Difference (LSD) post-hoc comparisons revealed differences between the 200 nM/ MK group and the 0 nM/ MK and Random/ MK groups (p<0.05). The 200 nM/ MK group did not differ from the 200 nM/ Saline or 0 nM/ Saline groups.

Cumulative incorrect lever presses over the 30-min extinction session for each of the groups are shown in Figure 2B. A two-way ANOVA (group by 5-min time interval; Fig 1B) revealed no significant effects.

Immediately after the extinction test, 3 animals that were injected with 200 nM aptamer into the nucleus accumbens and MK-801 systemically and 3 animals that were injected with vehicle into the nucleus accumbens and MK-801 systemically were euthanized for immunohistochemistry (results below).

### Locomotor Activity: intra-accumbens aptamer does not impair locomotion

**Figure 3:**
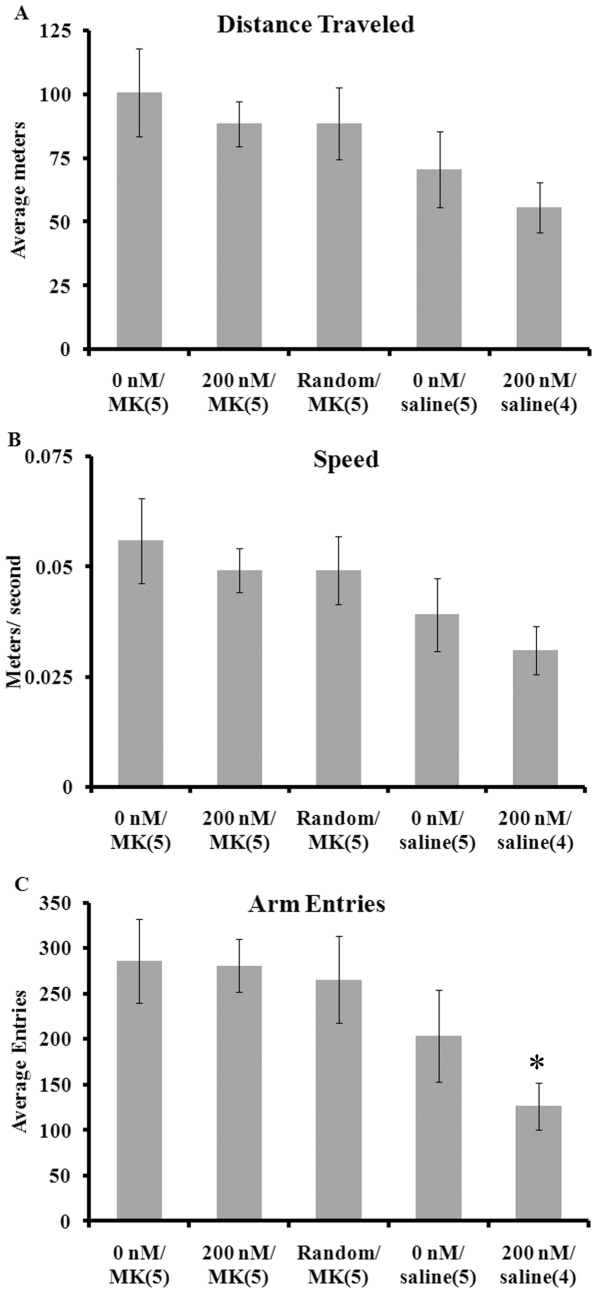
Horizontal activity was not affected by intra-accumbens injection of the aptamer. Locomotor activity was assessed in an elevated cross maze for 30 min. Activity was measured as total distance in meters traveled (A), average speed in meters/ sec (B) and total arm entries (C). No main effect of group was detected. Arm entries in all MK-801-injected groups (0 nM/ MK, 200 nM/ MK and Random/ MK; n = 5 per group) were significantly different from the 200 nM/ saline-treated group (n = 5) (*, p<0.05). The saline treated groups were not significantly different on any measure of activity. Data are expressed as mean ± SEM.

One week after the extinction test, the remaining animals were randomly re-assigned and given a second intra-accumbens injection and subjected to a 30-min locomotor test in a different apparatus. Data collected included distance traveled (in meters; Fig. 3A), speed (in meters/ sec; Fig. 3B) and arm entries (Fig. 3C). Data from 1 animal in the 200 nM/ Saline group were lost. Separate one-way ANOVAs were run on each of the dependent measures. For each measure, there were no main effects of group (Distance: F(4,19) = 1.53; Speed: F(4,19) = 1.53; Arm Entries: F(4,19) = 2.31).

### Histology/ Immunohistochemistry: Intra-accumbens aptamer injection reduces immunohistochemical staining of phosphorylated tyrosine hydroxylase

**Figure 4:**
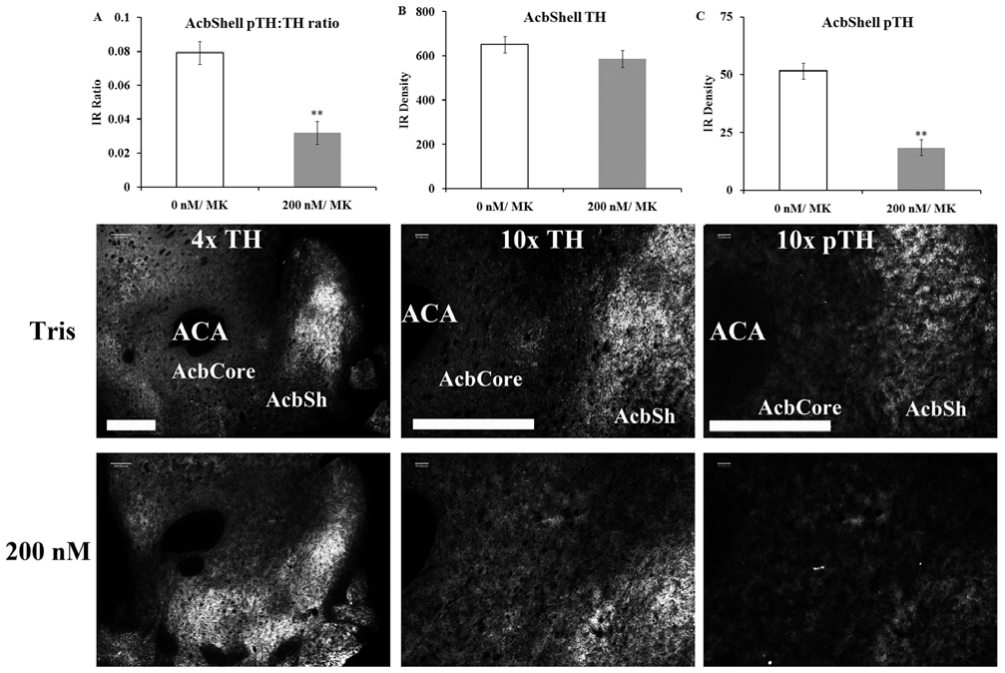
Representative histological sections from nucleus accumbens show reduced pTH staining in aptamer-treated rats. A) Quantification of the phosphorylated tyrosine hydroxylase (pTH) to tyrosine hydroxylase (TH) ratio in the nucleus accumbens shell region (AcbShell) shows a lower ratio in the 200 nM/ MK group (** p<0.01). B) Quantification of TH staining in the AcbShell shows similar levels between the 0 nM/ MK group and 200 nM/ MK group. C) Quantification of pTH staining in the AcbShell shows lower levels of staining in the 200 nM/ MK group compared to the 0 nM/ MK group (** p<0.01). n = 3/ group. Abbreviations: ACA, anterior commisure; AcbCore, nucleus accumbens core; AcbShell, nucleus accumbens shell. Scale bars = 500 µm. Magnifications shown at top of 0 nM images. Data expressed as mean ± SEM.

Fifteen minutes after the extinction test, 3 animals injected with 200 nM aptamer and 3 injected with vehicle into the nucleus accumbens (all injected systemically with MK-801) were euthanized and brains were removed for immunohistochemical analysis of tyrosine hydroxylase (TH) and phosphorylated TH (pTH) in the nucleus accumbens (Figure 4). The ratio of pTH: TH accumbens staining (Fig. 4A) was reduced in the 200 nM-injected group compared to the vehicle-injected group (t(4) = 7.02, p<0.01). This was not due to differences in TH staining (Fig. 4B; t(4) = 1.70) but rather due to reductions in pTH staining in the 200 nM group (Fig. 4C; t(4) = 9.25, p<0.01). Representative sections (Fig. 4D and E) from both groups show that the reduced pTH staining was localized to the nucleus accumbens shell region in the 200 nM-injected group.

## Discussion

A behavioural procedure to potentially model cognitive deficits associated with schizophrenia concerns the use of MK-801 during extinction of a learned response. A moderate dose (0.05–0.1 mg/kg) of MK-801 has been shown to produce a persistent, elevated lever pressing response throughout an entire 30 min extinction session [8]. MK-801 has been reported to increase dopamine output in the striatum [22] and frontal cortex [23] likely by increasing firing rates of mesolimbic dopaminergic neurons [24]–[25]. Consistent with this, animals pretreated with combined or separate dopamine D1- or D2-like receptor antagonists showed normalized extinction responding under MK-801 [8].

These findings can best be put into the context of a model concerning neural mechanisms underlying schizophrenia as developed by Carlsson and colleagues (see for example [26]–[27]. In this model, cortical regulation of dopamine output is mediated by two pathways: the “accelerator” and the “brake”. The brake pathway consists of combined glutamate and GABA synapses. Blockade of the glutamate portion of this pathway (for example, with MK-801) reduces GABA inhibitory control of midbrain dopaminergic neurons causing elevated dopamine output at terminal projection sites (e.g., nucleus accumbens, cortex). The work of Holahan et al., showed that MK-801 administration was associated with reduced neural activation in the infralimbic cortex and elevated extinction pressing that was blocked by dopamine receptor antagonists [8]. Therefore, MK-801 may increase rates of midbrain dopamine neural activity by reducing cortical glutamaterigic control leading to enhanced dopamine-dependent behaviors such as perseveration.

An alternative interpretation for the behavioral effect of MK-801 is that it altered a more fundamental motivational state enhancing the conditioned reinforcing properties of the conditioned cues. Indeed, MK-801 has been shown to prolong progressive ratio responding compared to saline-injected controls [8] and MK-801 potentiates both food unconditioned [28]–[29] and conditioned [30] behavioral responses. This could occur for a number of reasons including elevations in dopamine efflux in the Acb [8], [31], [32], a deficit in signaling processes that participate in termination of eating-satiety signals [29], or, as hypothesized in the present report, induction of a non-specific preservative effect on ongoing behavior. In any case, the behavioral changes observed can be ascribed to elevations in dopamine output in the Acb; the target of the aptamer injections.

In the current report, rats treated with 0.1 mg/ kg MK-801 systemically and either tris-buffer vehicle (0 nM group) or the random oligonucleotide sequence into the nucleus accumbens showed higher rates of lever pressing during extinction than rats treated systemically with saline (0 nM or 200 nM aptamer into the accumbens). The MK-801- injected group pre-treated with a 200 nM dose of the aptamer into the nucleus accumbens pressed significantly less than the MK-801 groups that received vehicle (0 nM) or the random oligonucleotide sequence pre-treatment in the nucleus accumbens. Furthermore, there were no significant difference in extinction pressing between groups that received central 0 nM or 200 nM aptamer pre-treatment and saline, rather than MK-801, peripheral administration. This, in conjunction with the finding that the 200 nM dose of aptamer did not affect incorrect lever pressing or locomotor activity, suggests that aptamer pre-treatment was selective in reversing, or at least minimizing, the cognitive-behavioral deficits of peripheral MK-801 administration.

The cross maze used to test locomotion was not one with open and closed arms as would be used to test anxiety but rather, one with all closed arms minimizing anxiety-eliciting effects. With the cross maze, both automated measures and manual measurements of arm entries were obtained. For all measures of locomotion, no differences were detected between MK-801-injected groups with either 0 nM, 200 nM or random aptamer injections. Horizontal activity in MK-801-injected animals is robustly elevated [8], [33]–[35] independent from the apparatus used. As there was no difference in activity between the groups injected with MK-801, it is concluded that the effects on extinction cannot be ascribed to a general lack of activity. While the 200 nM group injected with saline did show some reductions in the number of arm entries, this was not significantly different from the 0 nM/ saline group. Therefore, it would be premature to conclude that the aptamer by itself reduces locomotor activity.

Immunohistochemical staining of tyrosine hydroxylase (TH) and phosphorylated TH (pTH) in the nucleus accumbens showed reduced levels of pTH in the aptamer-injected animals compared to vehicle (both treated with MK-801). The synthesis and secretion of dopamine is critically dependent on TH activation through phosphorylation taking place at serine residues (Ser) 8, 19, 31 and 40 [36]. There is strong evidence that phosphorylation of tyrosine hydroxylase at Ser40 leads to TH activation and catecholamine (CA) synthesis *in vivo* and likely serves as a choke-point for dopamine and norepinephrine synthesis [36]. The observation that there was less immuno detection of phospho Ser40 TH suggests that not only does the aptamer bind to and render dopamine/norepinephrine dysfunctional but it may also limit the subsequent synthesis of dopamine/ norepinephrine. In this case, reduced pTH staining in the aptamer group would be indicative of a reduction in dopamine synthesis.

The reduction in pTH in the aptamer group might be due to low concentrations of free extracellular dopamine (i.e., not bound by the aptamer) working on presynaptic autoreceptors [37]. Consistent with this hypothesis, low concentrations of dopamine (DA) agonists, such as apomorphine (Apo), inhibit TH activity in striatal slices and synaptosomal preparations (reviewed by [37]. Initially, the aptamer would bind to dopamine/ norepinephrine that has been released into the synaptic cleft. When the aptamer is bound, it would reduce the effect of the neurotransmitter on the postsynaptic receptors. This is the primary/ direct effect of the aptamer that would happen immediately and for some unknown time. Binding of the aptamer to the target also has a secondary effect; in this case, reducing pTH and ultimately, CA synthesis. Therefore, the behavioral results are consistent with the primary/ direct effects of the aptamer and the secondary/ indirect effects of the aptamer (reduction in pTH). It should be noted that TH staining was similar between groups suggesting the aptamer did not damage dopaminergic terminals.

Results indicate that 200 nM aptamer pre-treatment was successful in instating extinction in hypoglutamatergic/hyperdopaminergic animals. If the failure of achieving extinction in MK-801-treated animals is partly caused by an overactivation of the mesolimbic dopaminergic system, the aptamer was successful in binding to dopamine within the nucleus accumbens, rendering it dysfunctional, resulting in a “normal” extinction process.

The strength of aptamer-target binding, which is the result of a combination of hydrogen bonding, electrostatic interactions, van der Waals forces and stacking interactions [38], is determined by the dissociation constant (K_d_). The judicious choice of an aptamer based on its binding affinity could allow for significant target binding, and therefore noticeable effects, only when dopamine concentrations are well above basal levels. The aptamer used in this study has K_d_ values in the hundreds of nanomolar for both dopamine and norepinephrine. As the basal dopamine level in the nucleus accumbens can be estimated to be 50 to 70 times less than this K_d_ [39], aptamer-target binding can be predicted to be very low. This may explain why no behavioural differences were noted in non-MK-801-treated rats. In MK-801-treated rats, dopamine concentrations in the Acb have been shown to increase by 135–145% [40]. Similar increases in dopamine output in the nucleus accumbens have also been reported during the presentation of cues which were previously paired with a food reward [41]. As comparable increases in dopamine are thought to be occurring in our extinction experiment, dopamine concentration, although still appreciably below the K_d_ of the aptamer, may perhaps be high enough to elicit more noticeable effects. Relative concentration arguments may also shed some light on the interaction of norepinephrine with this aptamer. Although this aptamer also binds with a comparable K_d_ to norepinephrine, basal and abnormal concentrations of norepinephrine in the nucleus accumbens are about an order of magnitude lower than the respective dopamine levels [40]. As a result, aptamer-norepinephrine complexes would be predicted to be considerably less abundant. Although this does not completely rule out a role for norepinephrine sequestration in these behavioral effects, it does suggest that, within this region of the brain at least, dopamine binding is more likely the cause.

Results of the current study provide several promising starting points for further exploration of aptamer technology in the central nervous system. First and foremost, finding that the aptamer, when injected into the nucleus accumbens, does not have overtly devastating consequences on behavioural output is a major step. Second, the near-selective effect on reversing cognitive deficits without drastic motoric impairment lends exceptional support for the use of DNA aptamers in the further study of preclinical animal models of mental health disease. A clear impediment to the use of aptamers in the brain will be their efficient and targeted delivery into the brain. Aptamer blood-brain-barrier (BBB) bypass is necessary in order to investigate the compound's pharmacokinetical properties and consider it a feasible therapeutic agent. Future work will examine strategies for delivery of these aptamers across the BBB, such as the use of immunoliposomes, and will investigate the pharmacokinetics of this aptamer *in vivo*.

## Materials and Methods

### Ethics Statement

All animal procedures were in accordance with Canadian Council on Animal Care (CCAC) guidelines, the NIH Guide for the Use and Care of Laboratory Animals and approved by the Carleton University Animal Care Committee (AUP ID P09-16).

### Subjects

Male Long Evans rats (n = 31) were purchased from Charles River, St. Constant, Quebec, Canada and housed in groups of two in polycarbonate 48×26×20 cm cages prior to surgery and individually housed post surgery. The vivarium was temperature (21°C) and lighting (12-hour light/dark cycle; lights on at 0800) controlled. Rats were handled for five minutes each day for seven days, to minimize stress. Approximately ten days following the intracranial surgical procedure, rats were placed on a food restriction schedule for approximately 8 days, until each animal reached a target of 85% of its initial weight (250–300 g).

### Surgical procedure

Standard stereotactic rodent surgical procedures were used. Animals were anaesthetized with isofluorane and two stainless steel 12 mm guide cannula (25 Ga) were implanted bilaterally, targeting the nucleus accumbens (Acb) at the following coordinates relative to bregma: antero-posterior (AP) = −1.7, latero-medial (LM) = +/−1.5 and dorso-ventral (DV) = −6.0. Cannula were secured in place using dental cement. Obturators (32 Ga) were inserted in each cannula. A subcutaneous dose (0.2 ml) of the analgesic Metacam was administered immediately after surgery and again 12 and 24 h later.

### Drugs

The noncompetitive NMDA receptor antagonist, MK-801 (Sigma-Aldrich), was stored frozen as a stock solution of 1.0 mg/ml in 0.9% sterile saline. It was thawed and diluted to the working concentration (0.10 mg/ kg; pH = 7.4) with 0.9% sterile saline on the day it was to be used. The dose was based on previous reports [7], [42]–[44].

The DNA aptamer [21], as well as the random oligonucleotide used in this work, were prepared by standard phosphoramidite chemistry on a Bioautomation MerMade DNA synthesizer (see Figure 1 for the sequences). The sequences were purified by polyacrylamide gel electrophoresis and the masses were confirmed by ESI-MS.

### Operant Conditioning Procedure

#### Acquisition

Six operant conditioning chambers (Coulbourn Instruments; 30.5 cm W×25.5 cm D×30.5 cm H) housed in insulated casings were used. Upon pressing the left lever two times (FR2), the house light went off, the panel lights above the lever changed from red to green and the pellet dispenser released one 45-mg chocolate pellet (BioServe, New Jersey) into the hopper. Presses on the left lever were correct and presses on the right lever, incorrect. During the five-day acquisition phase, rats were placed into the chambers for 30 minutes each day. Cumulative lever presses were recorded.

#### Extinction

The extinction session was 30 min and occurred on the third day after the last reinforced acquisition day. No food reward was delivered in response to correct lever pressing but the houselight went off and the panel lights changed from red to green. Cumulative lever presses were recorded.

### Locomotor testing

A subset of rats (n = 25) were tested for locomotor activity 1 week after the extinction test. The apparatus was an elevated (75 cm), closed wooden cross maze (60×9 cm arms). Each animal was placed into the center of the maze for a 30 min test. Horizontal activity (distance traveled, speed and arm entries) was recorded using the HVS Image 2100 Plus tracking system (HVS Image Ltd, UK).

### Central administration

Obturators were removed and two stainless-steel injection cannula (13 mm, 32 Ga) connected to two 10 µl Hamilton syringes by polyethylene tubing were inserted into the guide cannula. Syringes were connected to an injection pump (Braintree Scientific, Inc.), programmed to deliver a 0.5 µl injection at a rate of 0.25 µl/min. The injection cannula were left in place for an additional 60 s. During microinjections, each animal was allowed to move freely in its home cage.

### Peripheral administration

Each animal was administered a subcutaneous injection of MK-801 (0.1 mg/ kg in a volume of 0.3 ml) or saline immediately after the accumbens injection.

The following groups were used: intra-accumbens injection of tris buffer and systemic MK-801 (0 nM/ MK, n = 7); intra-accumbens injection of 200 nM aptamer and systemic MK-801 (200 nM/ MK, n = 7); intra-accumbens injection of the random oligonucleotide sequence and systemic MK-801 (Random/ MK, n = 7); intra-accumbens injection of tris buffer and systemic saline (0 nM/ Saline, n = 5); intra-accumbens injection of 200 nM aptamer and systemic saline (200 nM/ MK, n = 5). All animals were run through the operant acquisition and extinction procedure and a subset (n = 5/ group) were tested for locomotor activity.

### Histology

To investigate the *in* vivo effects of aptamer injection into the nucleus accumbens, 3 animals injected with 200 nM aptamer and an s.c. injection of 0.1 mg/ kg MK-801 and 3 animals injected with tris buffer and an s.c. injection of MK-801 were euthanized immediately after the extinction test. Brains were immersion fixed in a 4% paraformaldehyde/ 0.01 M phosphate buffer solution (PB; pH 7.4) and used for immunohistochemistry using methods as described [45]. Incubation in the primary antibodies (1∶1000 mouse anti-tyrosine hydroxylase from Immunostar or 1∶500 rabbit anti-phosphorylated tyrosine hydroxylase (S40) from Abcam) occurred overnight at room temperature. Incubation in the secondary antibodies (1∶500 goat anti-rabbit 594 or 1∶500 goat anti-mouse 488; Molecular Probes) occurred for 2 h at room temperature. Sections were mounted on glass slides and coverslipped using Fluormount (Sigma). Alternate sections were stained with cresyl violet to verify cannula placements.

### Immunohistochemical Quantification

Using an Olympus BX61 microscope (Olympus Canada, ON), digital images of the nucleus accumbens core and shell regions were obtained (20×, NA 0.4; InVitro version 3.2.2; Media Cybernetics, MD) using the same exposure time to reduce photobleaching and equalize intensities across subjects. Pixel intensity maps were generated for both TH and pTH staining using the Image-Pro Analyzer version 6.2.1.491 (Media Cybernetics, MD). Briefly, 5 horizontal lines were generated across the entire nucleus accumbens region and intensity measures were collected beginning at the anterior commisure followed by the core and finally the shell. An average raw pixel intensity profile was generated for each section. For quantification, intensity measures obtained in the shell were normalized to intensity measures in the anterior commisure (core staining was negligible at the exposure times used). Statistical analyses (t-tests) between groups were made using these normalized intensities on TH intensity, pTH intensity and the ratio of pTH∶TH to account for any variation in staining or image processing.

## Supporting Information

Video S1 This rat was given an intra-accumbens injection of 200 nM dose of the dopamine-binding aptamer and 5 min later, given a systemic injection of MK-801 prior to the extinction session. The video shows a 30 sec clip of bar pressing behavior approximately 20 min after the start of the extinction session.
(WMV)

Video S2 This rat was given an intra-accumbens injection of vehicle (Tris buffer; 0 nM) and 5 min later, given a systemic injection of MK-801 prior to the extinction session. The video shows a 30 sec clip of bar pressing behavior approximately 20 min after the start of the extinction session.
(WMV)

## References

[1] Reichenberg A (2010). The assessment of neuropsychological functioning in schizophrenia.. Dialogues Clin Neurosci.

[2] Linden DE (2007). The working memory networks of the human brain.. Neuroscientist.

[3] Ragozzino ME (2007). The contribution of the medial prefrontal cortex, orbitofrontal cortex, and dorsomedial striatum to behavioral flexibility.. Ann N Y Acad Sci.

[4] Waford RN, Lewine R (2010). Is perseveration uniquely characteristic of schizophrenia?. Schizophr Res.

[5] Neill JC, Barnes S, Cook S, Grayson B, Idris NF (2010). Animal models of cognitive dysfunction and negative symptoms of schizophrenia: Focus on NMDA receptor antagonism.. Pharmacol Ther.

[6] Meltzer HY, Horiguchi M, Massey BW (2011). The role of serotonin in the NMDA receptor antagonist models of psychosis and cognitive impairment.. Psychopharmacology (Berl).

[7] van der Meulen JA, Bilbija L, Joosten RN, de Bruin JP, Feenstra MG (2003). The NMDA-receptor antagonist MK-801 selectively disrupts reversal learning in rats.. Neuroreport.

[8] Holahan MR, Clarke MJ, Hines DD (2010). Dopamine-mediated MK-801-induced elevation in food-based extinction responding in rats and associated changes in region-specific phosphorylated ERK.. Psychopharmacology (Berl).

[9] Mayer G (2009). The chemical biology of aptamers.. Angew Chem Int Ed Engl.

[10] Nimjee SM, Rusconi CP, Sullenger BA (2005). Aptamers: an emerging class of therapeutics.. Annu Rev Med.

[11] Tuerk C, Gold L (1990). Systematic evolution of ligands by exponential enrichment: RNA ligands to bacteriophage T4 DNA polymerase.. Science.

[12] Stoltenburg R, Reinemann C, Strehlitz B (2007). SELEX–a (r)evolutionary method to generate high-affinity nucleic acid ligands.. Biomol Eng.

[13] de Franciscis V, Esposito CL, Catuogno S, Cellai L, Cerchia L (2009). Aptamers as innovative diagnostic and therapeutic agents in the central nervous system.. CNS Neurol Disord Drug Targets.

[14] Rentmeister A, Bill A, Wahle T, Walter J, Famulok M (2006). RNA aptamers selectively modulate protein recruitment to the cytoplasmic domain of beta-secretase BACE1 in vitro.. RNA.

[15] Wang Y, Khaing ZZ, Li N, Hall B, Schmidt CE (2010). Aptamer antagonists of myelin-derived inhibitors promote axon growth.. PLoS ONE.

[16] Mendonsa SD, Bowser MT (2005). In vitro selection of aptamers with affinity for neuropeptide Y using capillary electrophoresis.. J Am Chem Soc.

[17] Ulrich H, Ippolito JE, Pagan OR, Eterovic VA, Hann RM (1998). In vitro selection of RNA molecules that displace cocaine from the membrane-bound nicotinic acetylcholine receptor.. Proc Natl Acad Sci U S A.

[18] Hess GP, Ulrich H, Breitinger HG, Niu L, Gameiro AM (2000). Mechanism-based discovery of ligands that counteract inhibition of the nicotinic acetylcholine receptor by cocaine and MK-801.. Proc Natl Acad Sci U S A.

[19] Wang J, Zhang P, Li JY, Chen LQ, Huang CZ (2010). Adenosine-aptamer recognition-induced assembly of gold nanorods and a highly sensitive plasmon resonance coupling assay of adenosine in the brain of model SD rat.. Analyst.

[20] Mannironi C, Di Nardo A, Fruscoloni P, Tocchini-Valentini GP (1997). In vitro selection of dopamine RNA ligands.. Biochemistry.

[21] Walsh R, DeRosa MC (2009). Retention of function in the DNA homolog of the RNA dopamine aptamer.. Biochem Biophys Res Commun.

[22] Ali SF, Newport GD, Bracha HS (1994). Phencyclidine and (+)-MK-801-induced circling preference: correlation with monoamine levels in striatum of the rat brain.. Neurotoxicol Teratol.

[23] Padilla-de la Torre M, Franco-Perez J, Santamaria A, Galvan S, Gonzalez E (2008). Effect of acetaldehyde on behavioral and neurochemical changes induced by MK-801 in rats.. Ann N Y Acad Sci.

[24] Zhang J, Chiodo LA, Freeman AS (1992). Electrophysiological effects of MK-801 on rat nigrostriatal and mesoaccumbal dopaminergic neurons.. Brain Res.

[25] Murase S, Mathe JM, Grenhoff J, Svensson TH (1993). Effects of dizocilpine (MK-801) on rat midbrain dopamine cell activity: differential actions on firing pattern related to anatomical localization.. J Neural Transm Gen Sect.

[26] Carlsson A, Waters N, Carlsson ML (1999). Neurotransmitter interactions in schizophrenia–therapeutic implications.. Biol Psychiatry.

[27] Carlsson A, Waters N, Holm-Waters S, Tedroff J, Nilsson M (2001). Interactions between monoamines, glutamate, and GABA in schizophrenia: new evidence.. Annu Rev Pharmacol Toxicol.

[28] Covasa M, Hung CY, Ritter RC, Burns GA (2004). Intracerebroventricular administration of MK-801 increases food intake through mechanisms independent of gastric emptying.. Am J Physiol Regul Integr Comp Physiol.

[29] Burns GA, Ritter RC (1997). The non-competitive NMDA antagonist MK-801 increases food intake in rats.. Pharmacol Biochem Behav.

[30] Yonghui L, Xigeng Z, Yunjing B, Xiaoyan Y, Nan S (2006). Opposite effects of MK-801 on the expression of food and morphine-induced conditioned place preference in rats.. J Psychopharmacol.

[31] Ahn S, Phillips AG (2007). Dopamine efflux in the nucleus accumbens during within-session extinction, outcome-dependent, and habit-based instrumental responding for food reward.. Psychopharmacology (Berl).

[32] Hironaka N, Ikeda K, Sora I, Uhl GR, Niki H (2004). Food-reinforced operant behavior in dopamine transporter knockout mice: enhanced resistance to extinction.. Ann N Y Acad Sci.

[33] Danysz W, Essmann U, Bresink I, Wilke R (1994). Glutamate antagonists have different effects on spontaneous locomotor activity in rats.. Pharmacol Biochem Behav.

[34] Loscher W, Honack D (1992). The behavioural effects of MK-801 in rats: involvement of dopaminergic, serotonergic and noradrenergic systems.. Eur J Pharmacol.

[35] Murschall A, Hauber W (2005). Effects of a systemic AMPA/KA and NMDA receptor blockade on pavlovian-instrumental transfer.. Psychopharmacology (Berl).

[36] Dunkley PR, Bobrovskaya L, Graham ME, von Nagy-Felsobuki EI, Dickson PW (2004). Tyrosine hydroxylase phosphorylation: regulation and consequences.. J Neurochem.

[37] Goldstein M (1984). Regulatory mechanisms of dopamine biosynthesis at the tyrosine hydroxylase step.. Ann N Y Acad Sci.

[38] Matsugami A, Kobayashi S, Ouhashi K, Uesugi S, Yamamoto R (2003). Structural basis of the highly efficient trapping of the HIV Tat protein by an RNA aptamer.. Structure.

[39] Willuhn I, Wanat MJ, Clark JJ, Phillips PE (2010). Dopamine signaling in the nucleus accumbens of animals self-administering drugs of abuse.. Curr Top Behav Neurosci.

[40] Yan QS, Reith ME, Jobe PC, Dailey JW (1997). Dizocilpine (MK-801) increases not only dopamine but also serotonin and norepinephrine transmissions in the nucleus accumbens as measured by microdialysis in freely moving rats.. Brain Res.

[41] Bassareo V, Di Chiara G (1999). Modulation of feeding-induced activation of mesolimbic dopamine transmission by appetitive stimuli and its relation to motivational state.. Eur J Neurosci.

[42] Pitts RC, Buda DR, Keith JR, Cerutti DT, Galizio M (2006). Chlordiazepoxide and dizocilpine, but not morphine, selectively impair acquisition under a novel repeated-acquisition and performance task in rats.. Psychopharmacology (Berl).

[43] Wozniak DF, Olney JW, Kettinger L, Price M, Miller JP (1990). Behavioral effects of MK-801 in the rat.. Psychopharmacology (Berl).

[44] Port RL, Seybold KS (1998). Manipulation of NMDA-receptor activity alters extinction of an instrumental response in rats.. Physiol Behav.

[45] Holahan MR, Honegger KS, Tabatadze N, Routtenberg A (2007). GAP-43 gene expression regulates information storage.. Learn Mem.

